# Icterus

**Published:** 1842-11-19

**Authors:** 


*Icterus.*—Dr. Boyd's pathological contributions contain an account of the necrological appearances occurring in two cases of female infants labouring under jaundice. In one a female three weeks old, which was merely brought into the institution for interment, and of which no history could be obtained, the liver was dark-coloured, the gall-bladder rather flaccid; it contained a little glairy mucus in its fundus, in its upper portion, and cystic duct; there was a considerable quantity of dark-coloured matter of the consistence of soft wax filling it, and completely blocking up the duct, sufficient to form a complete mechanical obstruction to the flow of the bile. In the other case, a female, eight days old, the liver was dark-colored, and rather congested; the gall-bladder filled with limpid mucus; no bilious matter in the intestines; no obstructions were detected in the bile ducts. The whole body was of a brigrt yellow color, and the internal membranes were tinged of the same color.—*Ibid*, July, 1842.

